# Effects of ceiling fan use on cardiovascular autonomic function during simulated indoor overheating in bed‐resting older adults

**DOI:** 10.1113/EP094066

**Published:** 2026-09-03

**Authors:** Fergus K. O'Connor, Robert D. Meade, Kristina‐Marie T. Janetos, Norman R. Morris, Glen P. Kenny

**Affiliations:** ^1^ Human and Environmental Physiology Research Unit, School of Human Kinetics, Faculty of Health Sciences, Montpetit Hall University of Ottawa Ontario Canada; ^2^ School of Health Sciences and Social Work Griffith University Gold Coast Queensland Australia; ^3^ Australian Centre for Precision Health and Technology Griffith University Gold Coast Queensland Australia; ^4^ Griffith Institute for Human and Environmental Resilience Griffith University Gold Coast Queensland Australia; ^5^ Department of Epidemiology T.H. Chan School of Public Health Harvard University Boston Massachusetts USA; ^6^ Clinical Epidemiology Program Ottawa Hospital Research Institute Ottawa Canada

**Keywords:** cardiac function, extreme heat event, heat stress, thermoregulation, NCT06142890

## Abstract

Older adults are at heightened susceptibility to heat‐related cardiovascular complications when exposed to indoor overheating. While ceiling fans are recommended as an accessible cooling intervention, their effects on cardiac autonomic regulation remain unclear. In this randomized crossover study, 20 healthy older adults (median age 71 years, 12 female) completed two 8‐h exposures to simulated indoor overheating (31°C, 45% relative humidity) while bed‐resting with either no fan (control) or ceiling fan use (∼1.5 m/s airflow). Core temperature and heart rate were monitored. Before exposure and between hours 6 and 7, participants underwent cardiac autonomic response testing including measures of heart rate variability (SDNN, RMSSD – indices of overall and parasympathetic/vagal cardiac autonomic modulation, respectively) and supine‐to‐standing tests (30:15 ratio, systolic blood pressure response) as well as self‐reported symptoms of orthostatic intolerance. Ceiling fan use reduced peak core temperature (0.2°C [95% CI: 0.1–0.3]; *P *< 0.001), heart rate (−5 beats/min [−10 to 0]; *P* = 0.03), and attenuated heat‐induced reductions in RMSSD (4.7 ms [0.2–9.2]; *P* = 0.04) compared to control. However, fan use did not influence SDNN, blood pressure during postural changes, cardiac response to standing, or symptoms of orthostatic intolerance. While ceiling fans provided modest reductions in core temperature and heart rate and partially preserved RMSSD, they offered limited overall cardiovascular and autonomic protection in older adults during prolonged heat exposure. These findings indicate that, despite some beneficial effects, ceiling fans provide limited cardiovascular autonomic protection, underscoring the need for comprehensive cooling strategies to safeguard older adults during indoor overheating.

## Abstract (highlights)

- **What is the central question of this study?**
  Can ceiling fan use meaningfully preserve cardiac autonomic regulation and cardiovascular control in older adults during prolonged indoor heat exposure?
- **What is the main finding and its importance?**
  Ceiling fan use modestly reduced thermal and cardiac strain and partially preserved parasympathetic cardiac modulation during prolonged indoor heat exposure in older adults, but did not improve overall autonomic variability, blood pressure regulation or orthostatic tolerance. These findings demonstrate that small reductions in thermal strain do not translate to preservation of integrated cardiovascular control, highlighting the limitations of fan use as a standalone cooling strategy.

## 1 INTRODUCTION

Rising global temperatures are increasing the incidence of hot weather and the likelihood of indoor overheating and accompanying health risks (Kenny et al., 2024; O'Connor, Bach et al., 2025). Older adults (≥65 years), particularly those with cardiovascular diseases, are among the most susceptible to the dangers of environmental heat exposure (Kenny et al., 2024; Liu et al., 2022), primarily due to age‐related reductions in thermoregulatory function (Meade et al., 2020), compounded by impaired cardiac function (Liu et al., 2022; Meade et al., 2025).

While air conditioning and other forms of ambient cooling remain the most effective interventions for mitigating heat exposure in the home, access is limited for many individuals (Davis et al., 2021). As a result, alternative cooling interventions are commonly promoted (O'Connor, Kenny et al., 2025). Due to their ease of use and wide availability, electric fans are often highlighted as an accessible in‐home cooling intervention for older adults (Jay et al., 2021). However, despite older adults’ willingness to use fans during periods of indoor overheating (Oberai et al., 2026), previous laboratory and modelling studies have shown limited physiological benefits of pedestal fans when temperatures surpass 36–38°C (Chaseling et al., 2024; Meade, Notley et al., 2024; O'Connor et al., 2024). Extending these findings, we recently showed reductions in core temperature and heart rate that approached, but did not exceed, thresholds of clinical relevance (0.2°C core temperature reduction, 5 beats/min heart rate reduction) in older adults during daylong bed rest at 31°C with ceiling fan use (O'Connor et al., 2026). However, these modest reductions were not accompanied by improvements in blood pressure responses, rate–pressure product, or subjective symptoms of orthostatic tolerance during the transition from supine to standing during the 8‐h heat exposure (O'Connor et al., 2026). This raises important questions as to whether reductions in heart rate reflect meaningful alterations in cardiac autonomic control under conditions of indoor heat stress with potential implications for downstream cardiovascular risk.

Age‐related impairments in cardiac autonomic function may reduce cardiovascular adaptability during heat exposure, thereby reducing heat tolerance and increasing vulnerability to heat‐related cardiovascular events in older adults. Accordingly, determining whether accessible cooling strategies, such as ceiling fans, can meaningfully preserve autonomic function during prolonged indoor heat exposure is of considerable public health interest. In this secondary analysis, we evaluated whether ceiling fan use meaningfully preserves cardiac autonomic regulation in bed‐resting older adults exposed to daylong simulated indoor overheating.

## 2 METHODS

The study was approved by the University of Ottawa Research Ethics Board (H‐11‐21‐7575). The study conformed to the standard set by the *Declaration of Helsinki*, and was registered in a database (Clinical Trials identifier: NCT06142890). Verbal and written informed consent were obtained from all participants prior to commencing any experimental procedures.

Older adults aged 65–85 years from Ottawa, Canada, participated in this study. Experimental procedures are described in the supplemental materials of our previous report, with key details summarized here for context (O'Connor et al., 2026).

Participants completed two randomized 8‐h exposures to 31°C and 45% relative humidity, consistent with temperatures recorded indoors in Canada (Henderson et al., 2022). For the duration of the 8‐h heat exposure, aside from short breaks to use the bathroom, participants remained in a semi‐reclined position on a medical bed directly beneath a ceiling fan delivering air speeds of 0 (control) or ∼1.5 m/s (fan use). After 6 h, participants undertook a cardiac autonomic response test battery (described below). Drinking water (∼22°C) was available ad libitum throughout the exposure.

The primary outcome was peak core (rectal) temperature, where, a ≥0.2°C change was deemed clinically meaningful (O'Connor et al., 2024). While a sample of 17 participants was required to detect this effect with >90% power, this secondary analysis was not specifically powered for autonomic outcomes and should therefore be considered exploratory. Prespecified secondary outcomes included resting heart rate variability (standard deviation of normal to normal RR intervals [SDNN] and root mean square of successive differences between normal to normal RR intervals [RMSSD]), the cardiac response to standing (30:15 ratio), the systolic blood pressure response to standing from supine, and symptoms of orthostatic tolerance (sum of scores on 6 items; scale from 0 [no symptoms] to 10 [worst possible]) during the cardiac autonomic response test battery conducted at hour 6.

### 2.1 Outcome measurement and processing

#### 2.1.1 Core temperature

Rectal temperature was continuously monitored as an index of core temperature using a general‐purpose thermocouple temperature probe inserted ∼12 cm past the anal sphincter (Mon‐a‐therm General Purpose Temperature Probe, Mallinckrodt Medical Inc., Bridgewater, NJ, USA) throughout the duration of the heat exposure. Data were collected at a sampling rate of 1 kHz using LabChart physiological data analysis software (version 8.1.16.2019, ADInstruments, Dunedin, New Zealand) and downsampled to 5 s sampling intervals before analysis.

#### 2.1.2 Skin temperature

Skin temperature data were collected at 1‐min intervals using validated (Bach et al., 2026) temperature data loggers (DS1922L Thermochron, OnSolution Pty Ltd, Sydney, Australia) affixed to eight body regions using double‐sided adhesives and medical tape. Mean skin temperature was calculated according to the weightings recommended in ISO 9886:2004: 7% forehead, 17.5% right scapula, 17.5% upper left chest, 7% upper right arm, 7% right forearm, 5% left hand, 19% right anterior thigh and 20% left calf (International Standards Organization, 2004).

#### 2.1.3 Cardiovascular test battery

A cardiac autonomic response test battery was performed prior to the commencement of the heat exposure (baseline resting) in a thermoneutral environment (∼22°C) and at the completion of 6 h of heat exposure (31°C, 45% relative humidity). Participants were instrumented with a 3‐lead ECG and finger photoplethysmography sensor on the middle and ring fingers of the right hand for continuous measurement of beat‐to‐beat RR intervals and blood pressure, respectively. Participants remained supine for a minimum of 15 min before the start of each test battery.

### 2.2 Resting heart rate variability

Heart rate variability (HRV) was evaluated during supine rest where participants were instructed to breathe at a normal depth and follow in time with an electronic metronome set at 0.25 Hz (i.e., 15 breaths/min) for a total of 8 min. Following a 4‐min period of normal breathing, participants completed another 8 min of guided breathing. HRV indices including SDNN and RMSSD were calculated using LabChart HRV software (ADInstruments). SDNN is an indicator of overall HRV, while RMSSD primarily signals vagally mediated changes in heart rate (Shaffer & Ginsberg, 2017). Upon data processing, the cleanest 5‐min period of the paced breathing data (i.e., without premature ventricular contractions, other irregular heart beats or movement artefacts) was used for each breathing period and utilized within final analysis.

### 2.3 Supine to standing test

Participants were instructed to stand (from a resting supine position) and maintain a relaxed posture without speech or movement for 3 min. Two consecutive supine‐to‐standing tests were conducted (each preceded by >10 min of supine rest). The cardiac response to standing was assessed via the 30:15 ratio, derived from the longest RR interval around the 30th beat (within ±5 beats) divided by the shortest RR interval around the 15th beat post‐standing. The 30:15 ratio provides an index of vagally mediated heart rate modulation to postural changes (Spallone et al., 2011). The systolic blood pressure response to standing was calculated as the difference between supine and standing systolic blood pressure. Standing blood pressure was taken as the lowest value measured after 1 and 2 min of standing. The supine to standing difference in systolic pressure serves as an index of sympathetic activity involved in blood pressure homeostasis (Spallone et al., 2011). During the supine‐to‐standing test, a sling was placed on the arm bearing the photoplethysmography sensor to support the hand at the approximate level of the left ventricle (4th intercostal space).

#### 2.3.1 Orthostatic intolerance

After each test battery, participants were asked to report whether they experienced any symptoms of orthostatic intolerance (Kaufmann et al., 2012) (i.e., dizziness, lightheadedness, blurry vision) while completing the supine‐to‐standing tests. Participants rated symptoms of (1) lightheadedness; (2) vision problems; (3) generalized weakness; (4) fatigue; (5) trouble concentrating; and (6) head and/or neck discomfort on an 11‐point scale (0, no symptoms, to 10, most severe). An overall symptom severity score was derived by summing individual sub‐scale ratings (with one point added before statistical analysis).

#### 2.3.2 Fluid intake and losses

Nude body mass was measured to the nearest 10 g using a digital weighing scale before and after each experimental session (CBU150X, Mettler Toledo Inc., Columbus, OH, USA). Hourly fluid consumption and lunchtime food consumption was measured to the nearest gram using a digital kitchen scale (MK200, Mafiti, Shenzhen, China). Net fluid losses through sweat and urine were quantified as the change in nude body mass, corrected for consumed food and fluid intake. Average whole‐body sweat rate was estimated as the average hourly change in body mass corrected for urine output and food and fluid consumption.

### 2.4 Statistical analysis

All data were processed with R statistical software (version 4.2.0, R Core Team). The analyst was blinded to the participant ID, experimental condition and, for the assessment of cardiac autonomic modulation, time point (data from baseline and end‐exposure were blinded with a separate code). Data from the two cardiac autonomic response tests during each test battery were averaged prior to analysis. Data were analysed using baseline‐adjusted linear mixed‐effects models (two‐tailed α = 0.05) with R, version 4.2.0. Average thermal and cardiovascular strain were indexed by the mean core temperature and heart rate over the 8‐h exposure, which are mathematically equivalent to time‐normalized area under the curve (AUC) measures. Data were analysed using linear mixed‐effects models including condition as a fixed effect (fan vs. no fan) with baseline values included as covariates. A random intercept for participant was included to account for repeated measures. Homoskedasticity and normality of residuals (fixed and random effects) were evaluated via visual inspection of diagnostic plots (Lüdecke et al., 2021).

## 3 RESULTS

Of 38 individuals assessed for eligibility, 20 participated (60% female, median [interquartile range] age: 71 [68–73] years, Table 1).

**TABLE 1:** Physical characteristics of enrolled participants.

Variable	All participants (n = 20)
Age (years)	71 (68–73)
Sex (*n* (%))	
Females	12 (60%)
Males	8 (40%)
Height (cm)	167 (157–173)
Mass (kg)	73.9 (61.3–81.1)
Body mass index (kg/m^2^)^a^	25.1 (23.6–28.0)
Body surface area (m^2^)^b^	1.9 (1.6–1.9)
Smoking status (*n* (%))^c^	
Never	14 (70%)
Past	6 (30%)
Habitual physical activity (min/week)^d^	150 (120–393)
Types of physical activity (*n* (%))^d^	
Walking	14 (70%)
Jogging, biking, swimming, rowing	3 (15%)
Organized sports	3 (15%)
Peak aerobic power (${\dot{V}}_{O_{2}\mathrm{peak}}$) (mL/kg/min)^e^	23 (21–25)
ACSM ${\dot{V}}_{O_{2}\mathrm{peak}}$ percentile (%)^e^	10 (4–20)
Using prescribed medications (*n* (%))^f^	15 (75%)
Heat‐vulnerability‐linked medications (*n* (%))^f^	
Anti‐hypertensives	9 (45%)
Anti‐depressants	1 (5%)
Metformin	1 (5%)
Haemoglobin A_1c_ (%)	5.6 (5.3–5.9)

*Note*: Values are medians and interquartile range (IQR) or number of participants (%). ^a^Body mass index calculated as weight in kilograms divided by the square of height in metres. ^b^Body surface area estimated using the Du Bois equation: 0.20247 × height in meters^0.725^ × mass in kg^0.425^. ^c^Smoking status determined via participant self‐report. Prospective participants were excluded if they were currently smoking. All past smokers quit ≥15 years prior to participation. ^d^Participant self‐reported physical activity level determined via the Canadian Society for Exercise Physiology Get Active Questionnaire (GAQ). The types of physical activity performed were determined via the Kohl Physical Activity Questionnaire. ^e^Prospective participants’ peak aerobic power (${\dot{V}}_{O_{2}\mathrm{peak}}$) was assessed during an incremental cycling test to volitional fatigue. Volunteers were excluded if their ${\dot{V}}_{O_{2}\mathrm{peak}}$ exceeded the 50th percentile of age‐ and sex‐specific normative data published by the American College of Sports Medicine (ACSM). ^f^Participant prescription medication use determined via self‐report. Some participants reported taking medications that have been suggested to increase heat‐vulnerability (e.g., antidepressants) or are associated with health conditions known to reduce heat tolerance (e.g., type 2 diabetes, heart disease). Reported medications included: antihypertensives (e.g., ACE inhibitors, angiotensin II receptor blockers, diuretics, calcium channel blockers; *n* = 9), antidepressants (e.g., serotonin and noradrenaline reuptake inhibitors, selective serotonin reuptake inhibitors, *n* = 1), metformin (*n* = 1), statins (*n* = 8), topical creams/ointments (*n* = 2), corticosteroids (*n* = 4), bronchodilators (n = 1), medications treating hypoactive thyroid (*n* = 4), occasional pain (e.g., for headaches and joint pain; *n* = 3), and osteoporosis (*n* = 4).

### 3.1 Resting physiological outcomes

Peak (*P *< 0.001), end exposure (*P* = 0.01) and AUC (*P *< 0.001) core temperatures were lower with fan use compared with the control condition (Table 2). Similarly, peak (*P* = 0.03), end exposure (*P* = 0.03) and AUC heart rate (*P* = 0.02) were lower with fan use across the daylong heat exposure (Table 2). While peak skin temperature was lower with fan use (*P *< 0.001), there were no differences in end exposure (*P* = 0.05) and AUC skin temperature (*P* = 0.38) between trial conditions (Table 2).

**TABLE 2:** Outcome variables at baseline and at the end of the simulated indoor overheating exposure and between‐condition comparisons.

	Raw data, mean (SD)^a^		Fan–No fan^b^	
Variable	No fan	Fan	Difference (95% CI)	*P*
**Core temperature** (°C (pre, peak, end) or °C h over baseline (area under curve))				
Pre‐exposure	36.7 (0.3)	36.7 (0.3)		
Peak (primary)	37.8 (0.2)	37.6 (0.2)	−0.2 (−0.3 to −0.1)	<0.001
End exposure	37.6 (0.2)	37.5 (0.2)	−0.1 (−0.2 to 0.0)	0.01
Area under curve	5.3 (1.5)	4.1 (1.0)	−0.9 (−1.4 to −0.5)	<0.001
**Heart rate** (beats/min (pre, peak, end) or total beats over baseline (area under curve))				
Pre‐exposure	57 (6)	57 (6)		
Peak	84 (14)	79 (10)	−5 (−10 to 0)	0.03
End exposure	68 (8)	66 (8)	−2 (−4 to 0)	0.03
Area under curve	4782 (1916)	4111 (2020)	−720 (−1338 to −102)	0.02
**Skin temperature** (°C (pre, peak, end) or °C h over baseline (area under curve))				
Pre‐exposure	30.2 (0.5)	30.4 (0.5)		
Peak	35.6 (0.2)	35.1 (0.2)	−0.5 (−0.7 to −0.4)	<0.001
End exposure	35.2 (0.3)	34.8 (0.2)	−0.4 (−0.9 to 0.0)	0.05
Area under curve	85 (7)	83 (7)	−2 (−7 to 3)	0.38

^a^ Raw data represent mean and standard deviation of data measured prior to and during the last 15 min of the 8‐h exposure to 31°C and 45% relative humidity (and the pre‐post exposure change, ∆) in each of the control (no fan use), and ceiling fan experimental interventions for *n* = 20 participants (unless reported otherwise).

^b^ Between‐intervention mean differences and 95% confidence intervals (CI) analysed using a linear mixed‐effects model including fixed effects of cooling intervention (2 levels: Control (no fan use), fan use conditions) and baseline values (continuous covariate), with a random intercept for participant. For each outcome, 95% CI and *P*‐values for comparisons between trial arms are corrected for multiplicity using the Bonferroni method.

### 3.2 Cardiac autonomic responses

Following 6 h of heat exposure, fan use mitigated the heat‐induced reduction in RMSSD (mean difference: 4.7 ms [95% CI: 0.2 to 9.2]; *P* = 0.04; Figure 1). Despite this, fan use had no effect on SDNN (mean difference: 2.7 ms [95% CI: −0.9 to 6.3]; *P* = 0.14; Figure 1).

**FIGURE 1:**
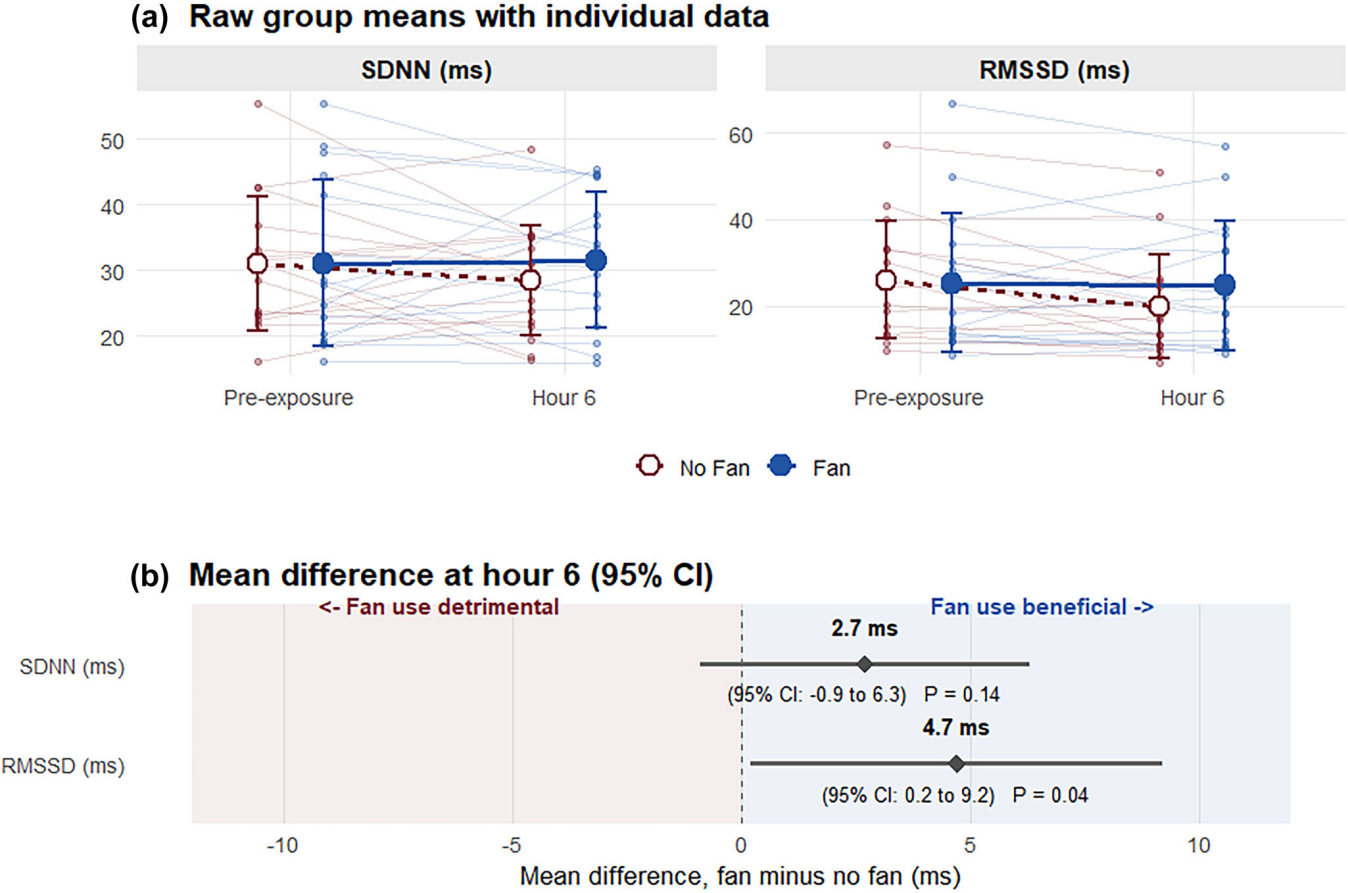
Influence of ceiling fan use during daylong heat exposure (31°C, 45% relative humidity) on SDNN and RMSSD recorded during a cardiac autonomic test battery at the conclusion of 6 h of exposure.

Cardiac and systolic pressure responses to standing were not different between conditions. Specifically, the 30:15 ratio at hour 6 was unaffected by fan use (mean difference: 0.02 [95% CI: −0.01 to 0.04]; *P* = 0.18; Figure 2), as was the systolic pressure response to standing (mean difference: 0 mmHg [95% CI: −3 to 4]; *P* = 0.77; Figure 2). Self‐reported symptoms of orthostatic intolerance scores were also not different between trial conditions (mean difference: −0.3 [95% CI: −0.7 to 0.1]; *P* = 0.12; Figure 2).

**FIGURE 2:**
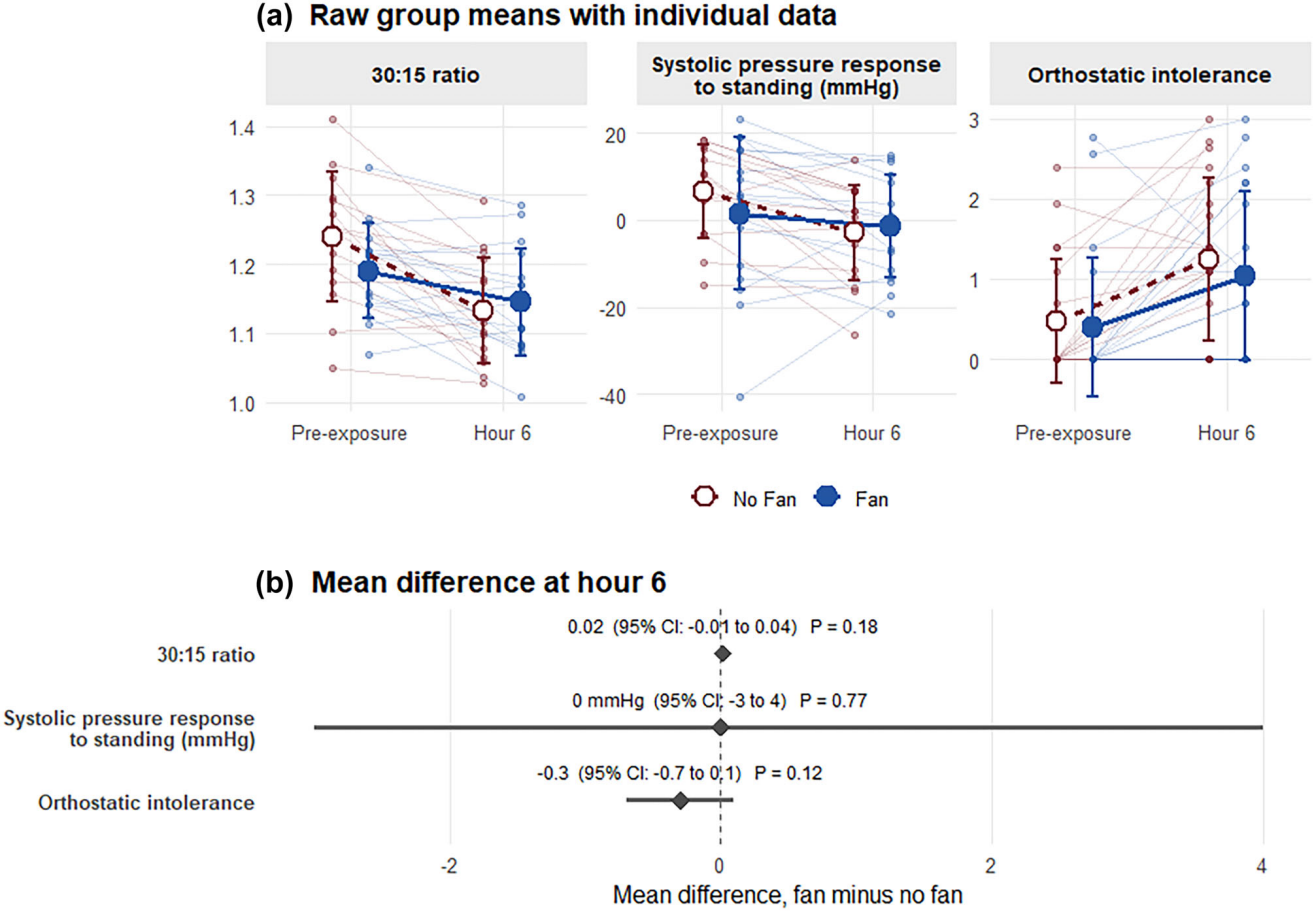
Influence of ceiling fan use during daylong heat exposure (31°C, 45% relative humidity) on the 30:15 ratio, systolic pressure response to standing and self‐reported symptoms of orthostatic intolerance recorded during a cardiac autonomic test battery at the conclusion of 6 h of exposure.

### 3.3 Fluid intake and losses

Fluid consumption was lower in the fan condition (mean difference: 23 mL/h [95% CI: 5 to 40]; *P* = 0.01). However, neither sweat rate (mean difference: −24 g/h [95% CI: −49 to 1], *P* = 0.06) nor net fluid loss (mean difference: 0.2 g/h [95% CI: −0.2 to 0.6]; *P* = 0.29) differed between trial conditions.

## 4 DISCUSSION

We examined whether ceiling fan use during prolonged indoor overheating preserves cardiac autonomic regulation in older adults. While ceiling fan use elicited modest reductions in core temperature and heart rate, approaching but not exceeding, thresholds of clinical relevance and attenuated reductions in RMSSD, fan use did not improve blood pressure regulation, symptoms of orthostatic tolerance and SDNN. Collectively, these findings suggest that although ceiling fans confer modest reductions in peak core temperature and heart rate, they do not appear to provide substantive autonomic protection during prolonged heat exposure in older adults.

In both laboratory (Meade, Akerman et al., 2024) and field (O'Connor, Bach et al., 2025) settings, heat stress consistently reduces heart rate variability in older adults, with the magnitude of reduction proportional to the thermal strain experienced (Meade, Akerman et al., 2024; O'Connor, Bach et al., 2025). In the present study, we observed that ceiling fan use attenuated the heat‐induced decline in RMSSD, a marker of parasympathetic cardiac modulation, suggesting a modest preservation of vagal tone during heat exposure. However, this effect did not translate to improvements in SDNN, which reflects overall autonomic variability and includes both sympathetic and parasympathetic contributions (Kane et al., 2025). Thus, the divergence between RMSSD and SDNN responses suggests that while ceiling fans may provide modest preservation of vagal modulation, they do not substantially alter overall autonomic balance during prolonged heat stress in older adults. However, further studies are likely required to elucidate these effects. Nonetheless, this interpretation appears to align with the modest reductions in core temperature and heart rate observed with fan use, which approached but did not exceed clinically meaningful thresholds (0.2°C core temperature, 5 beats/min heart rate). Together, these findings suggest that ceiling fans alone provide limited cardiovascular protection during prolonged indoor overheating in bed‐resting older adults, despite modest reductions in thermal strain.

Heat exposure impairs orthostatic tolerance through multiple mechanisms, including decreased blood volume, peripheral vasodilation and altered baroreflex sensitivity (Schlader et al., 2016). In the current investigation we found no differences in the cardiac response to standing (30:15 ratio), systolic blood pressure responses or self‐reported symptoms of orthostatic intolerance between trial conditions. These findings align with our previous work examining cardiac autonomic responses during prolonged heat stress in older adults with and without foot immersion and neck cooling interventions (McCourt et al., 2024), and suggest that the modest reductions in core temperature achieved with ceiling fans are insufficient to meaningfully preserve cardiovascular responses to postural challenges. Given older adults are already at heightened risk of falls and syncope (Saunders et al., 2025), the lack of improvement in self‐reported symptoms of orthostatic tolerance with fan use suggests ceiling fans offer limited protection against postural cardiovascular instability during heat exposure. These findings further support a dissociation between modest reductions in thermal strain and preservation of integrated cardiovascular control during orthostatic challenge.

### 4.1 Perspective and significance

Our findings have important implications for heat management within the home. While ceiling (and pedestal) fans are frequently recommended as an accessible cooling strategy (Jay et al., 2021), our data suggest fans provide only limited cardiovascular protection during prolonged indoor heat exposure at 31°C. In accordance with recent recommendations (Bone et al., 2025), our data suggest that where possible, ceiling fans should be used in conjunction with air‐conditioning to maintain indoor temperatures at ∼27°C (Meade, Notley et al., 2024), thereby providing adequate thermoregulatory and cardiovascular protection during heat events, while reducing electrical power consumption associated with air conditioner use. Although energy consumption was not evaluated in the present study, this combined approach has the potential to reduce cooling‐related energy demand compared with maintaining substantially lower indoor temperatures using air conditioning alone.

### 4.2 Limitations

First, as acknowledged in the methods, this analysis is secondary and exploratory in nature, and the study was powered to detect changes in core temperature rather than cardiac autonomic responses. Consequently, these findings should be interpreted with caution and require confirmation in adequately powered studies, ideally under free‐living conditions. In addition, heart rate variability was assessed only before heat exposure and after 6 h of exposure. Serial measurements throughout the exposure may have provided additional insight into the temporal evolution of cardiac autonomic responses. Second, participants remained in prolonged bed rest, which may underestimate the physiological demands experienced during normal daily activities during heat events. Third, we examined a single temperature and humidity combination (31°C, 45% relative humidity); therefore our findings may not fully reflect the dynamic nature of indoor heat exposure (Oberai et al., 2025). Although an 8‐h thermoneutral control condition was not included in the present study, previous work from our laboratory demonstrated that prolonged exposure to indoor temperatures between 22 and 26°C does not elicit the progressive increases in core temperature or cardiovascular strain observed at 31°C and above in older adults (Meade, Akerman et al., 2024). Finally, our sample consisted of healthy older adults free from cardiovascular diseases. As a result, individuals living with chronic conditions or frailty may exhibit divergent responses.

### 4.3 Conclusion

Ceiling fan use during prolonged indoor heat exposure at 31°C resulted in modest reductions in core temperature and heart rate and attenuated heat‐induced reductions in parasympathetic cardiac modulation (RMSSD) in older adults. However, fan use did not improve overall autonomic variability (SDNN), blood pressure regulation, or symptoms of orthostatic tolerance. These findings suggest that while ceiling fans provide modest thermoregulatory benefits, they offer limited cardiovascular and autonomic protection during prolonged indoor overheating. As indoor overheating becomes an increasingly common reality, these findings highlight a dissociation between reductions in thermal strain and preservation of cardiovascular autonomic function, indicating that reducing thermal strain alone is insufficient to ensure cardiovascular protection during heat exposure. Accordingly, ceiling fans should be used as part of a broader, integrated cooling strategy rather than as a standalone intervention to mitigate heat‐related cardiovascular risk in older adults.

## AUTHOR CONTRIBUTIONS

G. P. Kenny had full access to the data in the study and takes responsibility for the integrity of the data and the accuracy of the data analysis. Concept and design: O'Connor, Meade, Kenny. Acquisition, analysis, or interpretation of data: All authors. Drafting of the manuscript: O'Connor, Meade, Kenny. Critical revision of the manuscript for important intellectual content: All authors. Statistical analysis: Meade. Obtained funding: Meade, Kenny. Administrative, technical, or material support: Kenny. Supervision: Meade, Kenny. All authors have read and approved the final version of this manuscript and agree to be accountable for all aspects of the work in ensuring that questions related to the accuracy or integrity of any part of the work are appropriately investigated and resolved. All persons designated as authors qualify for authorship, and all those who qualify for authorship are listed.

## CONFLICT OF INTEREST

The authors declare they have no conflicts of interest.

## GENERATIVE AI STATEMENT

The lead author used Claude Sonnet 5 to check for grammatical errors within the manuscript. The suggested edits were verified prior to implementation within the manuscript.

## Data Availability

De‐identified participant data and supporting documentation will be made available from the corresponding author upon publication (Dr Kenny, gkenny@uottawa.ca). All data requests should include a detailed re‐analysis plan and will require a signed access agreement.
